# Substitution Effect on the Charge Transfer Processes in Organo-Imido Lindqvist-Polyoxomolybdate

**DOI:** 10.3390/molecules24010044

**Published:** 2018-12-22

**Authors:** Patricio Hermosilla-Ibáñez, Kerry Wrighton-Araneda, Walter Cañón-Mancisidor, Marlen Gutiérrez-Cutiño, Verónica Paredes-García, Diego Venegas-Yazigi

**Affiliations:** 1 Facultad de Química y Biología, Universidad de Santiago de Chile, USACH, Av. Libertador Bernardo O’Higgins 3363, 9170022 Santiago, Chile; patricio.hermosilla@usach.cl (P.H.-I.); kerry.wrighton@usach.cl (K.W.-A.); walter.canon@usach.cl (W.C.-M.); marlen.gutierrez@usach.cl (M.G.-C.); 2 Centro para el Desarrollo de la Nanociencia y Nanotecnología, CEDENNA, 9170022 Santiago, Chile; 3 Departamento de Ciencias Químicas, Universidad Andres Bello, Republica 275, 8370146 Santiago, Chile; vparedes@unab.cl

**Keywords:** organoimido, polyoxometalate, charge transfer, electronic properties, TD-DFT, Rhenium

## Abstract

Two new aromatic organo-imido polyoxometalates with an electron donor triazole group ([*n*-Bu_4_N]_2_[Mo_6_O_18_NC_6_H_4_N_3_C_2_H_2_]) (**1**) and a highly conjugated fluorene ([*n*-Bu_4_N]_2_[Mo_6_O_18_NC_13_H_9_]) (**2**) have been obtained. The electrochemical and spectroscopic properties of several organo-imido systems were studied. These properties were analysed by the theoretical study of the redox potentials and by means of the excitation analysis, in order to understand the effect on the substitution of the organo-imido fragment and the effect of the interaction to a metal centre. Our results show a bathochromic shift related to the charge transfer processes induced by the increase of the conjugated character of the organic fragment. The cathodic shift obtained from the electrochemical studies reflects that the electronic communication and conjugation between the organic and inorganic fragments is the main reason of this phenomenon.

## 1. Introduction

Polyoxometalates (POMs) represent a large family of anionic clusters formed by oxygen and transition metal atoms presenting a rich structural and electronic versatility [1,2,3]. Among the family of POMs, the Lindqvist-type hexamolybdate [Mo_6_O_19_]^2−^ is one of the most studied [4,5,6] due to its simplicity and the high symmetry between the six centres. The functionalization of this compound with organo-imido fragments has a general formula [Mo_6_O_(19−x)_(N-R)_x_]^2−^, where x = 1–6 and N-R is an organic unit derived from a isocyanate or a primary amine molecule bonded to a molybdenum centre [7,8,9]. This functionalization permits to obtain and design new organic-inorganic hybrid systems where the electronic properties can be modulated depending on the nature of the incorporated organic fragment [10].

The charge transfer phenomenon from an electron-rich fragment to an acceptor fragment, both covalently bonded, is an interesting feature that these molecular systems can present. The ligand to metal charge transfer (LMCT) transition in the hexamolybdate, i.e., from the oxygen to the molybdenum centres, is in the region of ≈325 nm. Upon functionalization with organic fragments, an intense band with a maximum at lower energy is observed. For long time it was assigned to a shift of the LMCT (oxygen to molybdenum) [11]. However, according to the results published by our group the band that appears at lower energies is a new band that corresponds to LMCT from the organic fragment to the metal centres [12]. The use of theoretical calculations based on TD-DFT (time-dependent density functional theory) has permitted the corroboration of experimental assignments of these bands, as reported by Ravelli et al. [13].

The electrochemical processes have also been studied, both theoretically and experimentally. From the theoretical point of view, the work published by López et al. recommends the use of a different approach to calculate redox potentials of polyoxometalates [14] with respect to the method published by Truhlar et al. [15].

As the frontier orbitals are involved in the electrochemical and electronic properties of these hybrid materials, the nature of the organic fragment will affect both of them. The electronic features of the organic fragment can be tuned through the introduction of organic substituents and/or the bonding of coordination compounds. It is possible to find some examples of this kind of functionalization with first, second, and third row transition metal complexes [12,16,17,18,19,20].

It has been shown that the presence of an isolated and well-defined charge transfer (CT) transition is necessary to have good charge transfer process. For example, it has been reported that a ferrocenyl unit, which is an electron-rich system, coordinated to an imido-functionalized hexamolybdate through a highly conjugated bridge, presents a charge transfer from the ferrocenyl to the hexamolybdate fragment more than 200 nm lower in energy than the oxygen to molybdenum CT of the hexamolybdate [17]. 

In this work, two new aromatic organo-imido polyoxometalates with an electron donor triazole group ([*n*-Bu_4_N]_2_[Mo_6_O_18_NC_6_H_4_N_3_C_2_H_2_]) (**1**) and a highly conjugated fluorene ([*n*-Bu_4_N]_2_[Mo_6_O_18_NC_13_H_9_]) (**2**) are presented. Five aliphatically-functionalized hexamolybdates were taken from the literature [21]. Seven aromatic systems with different electron donating and withdrawing abilities were also taken from the literature [10,22,23]. For the spectroscopic study, some molecules being the most representative systems were used. The electrochemical and spectroscopic properties of all systems were studied. These properties were analysed and discussed by means of the excitation analysis depending on the substitution of the organo-imido fragment and the effect of the interaction to a metal centre.

## 2. Results and Discussions

### 2.1. Synthesis and X-ray Structures

**Figure 1:**
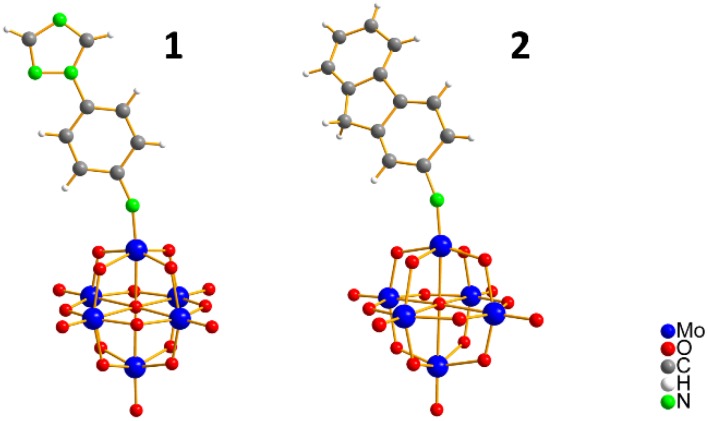
Ball-and-stick representation of **1** and **2** organo-imido-polyoxometalate. [n-Bu_4_N]^+^ ions are omitted for clarity.

Two new organo-imido polyoxometalates have been synthesized as described in Section 3. The compounds were crystalized, and the X-ray data are shown in the supporting information (Figures S1 and S2, and Table S1), given a formula of [*n*-Bu_4_N]_2_[Mo_6_O_18_NC_6_H_4_N_3_C_2_H_2_] for (**1**) and [*n*-Bu_4_N]_2_[Mo_6_O_18_NC_13_H_9_] for (**2**) (Figure 1). The analysis of the bond angle Mo-N-R gives an idea of the double/triple character of the Mo-N bond. The values observed in this work for **1** and **2**, as well as the literature values for the triazolylmethyl analogue of **1** (compound **3**) and four other compounds with aliphatic substitution (ethyl, n-propyl, n-butyl, n-hexyl) are summarized in Table 1.

It can be observed that all aliphatically-substituted compounds show angles with values higher than 176 degrees, i.e., showing the more linear structure, thus having the nitrogen with a more *sp* character. On the other hand, compounds with aromatic substituents have narrower Mo-N-C angles, hence nitrogen atoms with less triple-bond character toward molybdenum. Summarizing, in the case of aliphatic substituents, a more *sp* nitrogen atom is observed, therefore it interacts as a triple bond to the molybdenum atom and single bond to the substituent. In the case of the aromatic substitution, the interaction has a less of a triple-bond character to the molybdenum, and an increased double-bond interaction to the substituent.

### 2.2. Electrochemical Properties

Cyclic voltammetry studies of [*n*-Bu_4_N]_2_[Mo_6_O_18_NC_6_H_4_N_3_C_2_H_2_] (**1**) and [*n*-Bu_4_N]_2_[Mo_6_O_18_NC_13_H_9_] (**2**) were carried out in acetonitrile solution using *n*-Bu_4_NClO_4_ as a supporting electrolyte (Figure S3). In order to compare our values (**1** and **2**) with those obtained from the literature, the concept of cathodic shift (E_1/2_) is used. This shift is the difference between the E_1/2_ from the hexamolybdate and the E_1/2_ of each compound measured in the same conditions. The calculated cathodic shift (E_c_) was obtained in the same way, i.e., as the difference between the calculated E_1/2_ for each molecule and the E_1/2_ for the naked hexamolybdate. The use of ΔE_C_ and ΔE_1/2_ values allowed us to analyse the data independently of the reference electrode. The E_1/2-Teo_ of the hexamolybdate used to evaluate all the theoretical cathodic shifts is −0.687 V. The E_1/2_ used to calculate the ΔE_1/2_ for **1** and **2** was −0.836 V. Table 2 summarizes the experimental and theoretical cathodic shift for all studied compounds.

**Figure 2:**
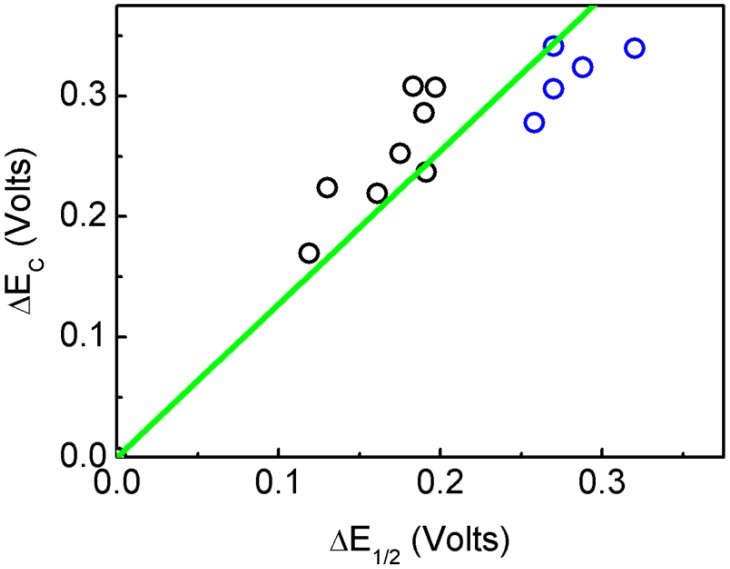
Relation between the experimental ΔE_1/2_ and theoretical ΔE_C_ cathodic shift potentials. In black the points correspond to the aromatic organo-imido POM and in blue the aliphatic ones.

A good correlation between the experimental and theoretical cathodic shift values, ΔE_1/2_ and ΔE_C_ respectively, have been obtained, giving a coefficient of determination, R^2^ = 0.9728 (Figure 2). The results show that the systems can be classified in two groups, aliphatic and aromatic. No clear relation between the length of the aliphatic chain and the redox potential is observed. On the other hand, the aromatic substitution affected the redox potential of the POM ranging from 0.119 to 0.197 V for the p-nitrophenyl group and the m-chlorophenyl group, respectively. Thus the ΔE_C_ depended on the ability of the organic fragment to increase the electron density into the POM. The most electron-withdrawing group, i.e., the nitro substitution, allowed for the POM fragment to be easily reduced. Moreover, the halogen substitution, which is an electron donor group, caused a more difficult reduction of the POM. By comparing **1** and **3,** it was possible to observe that compound **1** had a higher cathodic shift, probably due to the absence of the methylene group between the aromatic rings interrupting the electronic communication from the electron donating triazole group to the phenyl group (Figure S4). Finally, the cathodic shift of the Re^I^ complex of **3** is lower than that of compound **3** alone, meaning that this coordination compound was acting as a withdrawing group. These results suggest that organic functionalization affected the virtual orbitals, therefore shifting the redox potentials of these systems. It is interesting to point out that, as shown in Table 1, all aliphatic substituted systems show the Mo–N–C angle nearer 180°contrary to the aromatic ones, i.e., the aromatic substituents showed less of a triple-bond character between Mo and N, and an increased double-bond character between N and C, thus the aromatic system could receive electron density from the POM, lowering the reduction potential of the inorganic fragment.

### 2.3. Spectroscopic Characterization

**Figure 3:**
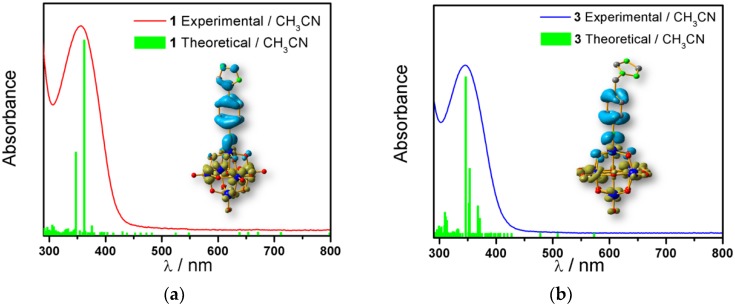
Overlap plot of the experimental spectra and oscillator strength calculated using TD-DFT methods, employing CH_3_CN as a continuum solvent. Hole–electron density surface for the most intensive excitation of: (**a**) compound **1** and (**b**) compound **3**. Light cyan and light brown represent the hole and electron surfaces, respectively.

UV-vis absorption spectra in acetonitrile solution of compounds **1** and **2** showed the lowest energy maximum at 356 nm and 386 nm, respectively (Figure 3 and Figure S5). This absorption band corresponded to the overlap of the oxygen to molybdenum charge transfer transition and the organic fragment to the molybdenum CT.

Theoretical calculations were done in order to understand the electronic spectra of this organo-imido systems using eight representative compounds. We have considered four aromatic systems [Mo_6_O_18_N-R]^2−^, where R = –C_6_H_5_ (**8**), –C_6_H_4_–N_3_C_2_H_2_ (**1**), –C_13_H_9_ (**2**) and –C_6_H_4_–CH_2_–N_3_C_2_H_2_ (**3**), two Re^I^ complexes, [Mo_6_O_18_NC_6_H_4_-CH_2_-N_3_C_2_H_2_-phenRe(CO)_3_]^−^ (**4**) and [Mo_6_O_18_NC_6_H_4_-N_3_C_2_H_2_-phenRe(CO)_3_]^−^ (**5**), and finally, two aliphatic systems [Mo_6_O_18_N–CH_2_CH_3_]^2−^ (**6**), and [Mo_6_O_18_N–CH_2_CH_2_CH_3_]^2−^ (**7**) in order to show how these groups can affect the charge transfer process. Using TD-DFT calculations, we were able to identify the oxygen (O) to molybdenum CT and the organic fragment (O.F.) to molybdenum CT.

The hole–electron formalism applied to molecular systems was employed to offer an efficient tool to assign each band, because this scheme takes into account the local and non-local contribution of the set of molecular orbitals involved in each excited state [12,24]. The hole-density surfaces represent electron density allocated in the ground state before it is to be promoted to an excited state (“hole”, light-cyan). The electron-density surface represents the electron density promoted in the vertical Franck–Condon excited state (“electron”, yellow), thus describing the excited state.

A band is formed via several transitions, and the transition presenting the higher oscillator strength value was taken as the representative. The most intense O.F. to Mo CT bands are presented in Table 3 and Table S2.

The calculation results show that the band related to the oxygen to molybdenum CT for all studied compounds was practically unaffected, appearing at the same energy of ≈311 nm. The molecular orbitals related to the charge transfer transition from the oxygen to molybdenum centres involved the d_xy_ orbitals. Therefore, these orbitals were not affected by the formation of the imido compound. In the case of the aliphatic systems, the oscillator strength related to the oxygen to molybdenum CT is more intense than the of organic fragment to molybdenum CT, as can be observed in Table 3 and Table S2. In the case of the oxygen to molybdenum CT for aromatic systems the value of the oscillator strength was around ten times lower than the of organic fragment to molybdenum charge transfer. On the other hand, when we compared the CT energy from the organic fragments to the molybdenum, it could be observed that fluorene was the one with the lowest energy absorption, whereas the aliphatic-substituted ones were those with highest energy absorption. Therefore, it can be inferred that a bathochromic shift exists, with this being related to the electron density given by the π-donor character of the organic fragment to the POM, decreasing the energy difference between the ground and excited states. All these results suggest that when the organic fragment is more conjugated, the O.F. to molybdenum CT band shifted to lower energies.

The molecular structure of compound **3** differed from **1** only in the existence of the methylene group between the phenyl and triazole fragments [12]. In **1**, the CT band appeared at lower energies (375 nm) compared to **3**, in which the CT energy absorption was at 346 nm. The existence of the methylene group in **3** disrupts the conjugation between phenyl-imido-POM and the triazole ring. The orbital structure based on frontier molecular orbitals was studied. For both systems, the LUMO (Lowest Unoccupied Molecular Orbital) was located mainly on the Mo centres, resembling the d_xy_ orbitals, whereas the HOMO (Highest Occupied Molecular Orbital) was located over the organic fragment (Figure S6). In **1**, the distribution of the HOMO was allocated all over the aromatic fragment (phenyl and triazole units), which does not happen in **3**.

The hole-electron surfaces show that the electronic communication between both aromatic rings is highly conjugated in 1, confirming that the electron transfer process occurs from the whole aromatic fragment to the molybdenum centres. When a comparison was made between this system and **3**, it was possible to observe that the electron transfer occurred from the phenyl group to the POM, meaning that the methylene group (–CH_2_–) cuts the electronic communication between the POM and 1,2,4-triazole ring (Figure 3).

In order to understand how a coordination compound can affect the charge transfer processes between the organic fragment to the POM, we have considered two coordination compounds based on Re^I^. The first corresponds to [Mo_6_O_18_NC_6_H_4_–CH_2_–N_3_C_2_H_2_–phenRe(CO)_3_]^−^(**4**), and the second to [Mo_6_O_18_NC_6_H_4_–N_3_C_2_H_2_–phenRe(CO)_3_]^−^ (**5**). Complex **4** is an organometallic system based on **3** that has been previously reported by us [12], whereas compound **5** is a proposed complex that is based on **1** and has been calculated by DFT methods as described in Section 3. The major difference between **4** and **5** is the presence of the methylene group between the phenyl and triazole rings. Both systems allowed us to compare how the coordination compound could affect the charge transfer processes (O.F. → Mo and O → Mo) in the compounds.

The calculation results showed that the band related to the oxygen to molybdenum CT was unaffected by the presence of the Re^I^ complex, since the energy of this absorption for **4** and **5** were at ≈311 nm, which is the same energy observed for **1** and **3,** the corresponding non-metalated systems (See Table S2). The energy of the O.F. to Mo CT for **4**, compared to **3**, was only 1 nm shifted to higher energies (See Table 3). However, in the case of **1** and **5**, this energy difference was 9 nm, shifted to higher energies (See Table 3). These results suggest that the coordination compound fragment had a small effect in modifying the CT processes of organo-imido systems.

**Figure 4:**
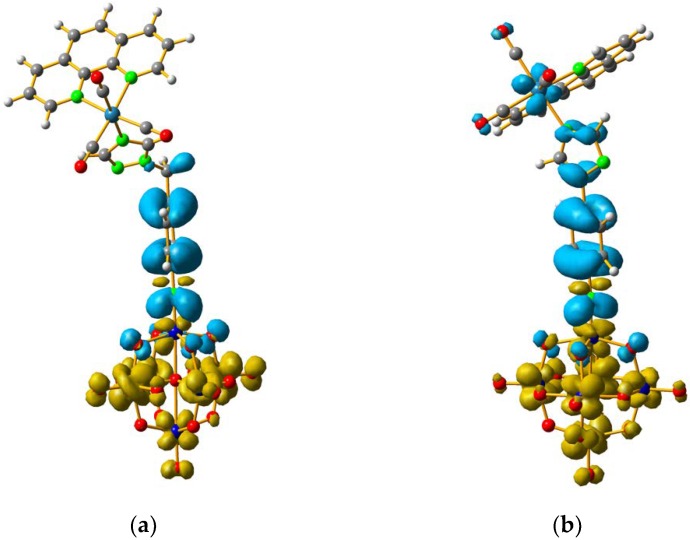
Hole-electron surfaces for complex **4** (**a**) and **5** (**b**) are presented. Light cyan and light brown represent the hole and electron surfaces, respectively.

For **4** and **5**, the LUMO orbitals were located mainly in the Mo centres, resembling the d_xy_ orbitals, as observed for **1** and **3**. The HOMO orbitals were located over the organic fragment, the Re^I^ centre and a small part in the CO ligands of the complex. The comparison between these frontier orbitals of **4** and **5** shows that there was a major distribution of these orbitals over the whole organic fragment, even over the Re^I^ centre in **5** (Figure S6). The hole–electron surface for **5** showed that the electron surface morphology was distributed all over the organic fragment and in the metal centres of the POM, which is quite similar to the d_xy_ orbital of the Mo ions. This characteristic could be due to the presence of the methylene group in **4**, meaning that in the case of **5**, the extension of the conjugation was between all the organic fragment of the organo-imido POM to the Re^I^ complex (Figure 4).

All these results suggested that when the organic fragment of an organo-imido POM system was more conjugated, the CT band moved to lower energies. The functionalization with a coordination compound produced a slight shift of this band to higher energies.

## 3. Materials and Methods 

### 3.1. Reagents and Instruments

All reagents were used without further purification. [*n*-Bu_4_N]_2_[Mo_6_O_19_] was synthesised according to a previously reported method in the literature [25]. The UV-vis spectra were obtained in acetonitrile at room temperature using a Perkin-Elmer Lambda 1050 Wideband UV-vis-NIR spectrophotometer (Perkin-Elmer, Waltham, MA, USA). FTIR-ATR (Fourier Transform Infrared- Attenuated Total Reflectance) spectra (4000–400 cm^−1^) of the compounds were measured using a Jasco FTIR-4600 spectrophotometer equipped with an ATR PRO ONE (Jasco, Easton, MD, USA). Elemental analyses (C, N, H) of bulk samples were performed using a Thermo elemental analyser Flash 2000. Electrospray ionization mass spectrometry (ESI-MS) studies were done for compounds **1** and **2** in a Thermo scientific Linear Ion Trap Mass Spectrometer LTQ XL. The mass detection was carried out using electrospray ionization (ESI), with the spray voltage set at 3 kV (at 250 °C). Detection was performed in full scan mode in the 100–1000 *m*/*z* range in negative mode. The spectra were obtained using He for collision-induced fragmentation (CID) with a normalized collision energy of 35 units and detection of fragments in full scan mode. Cyclic voltammetry (CV) studies were performed on a CH-Instruments 650E potentiostat. All electrochemical data were obtained in acetonitrile (*SeccoSolv*^®^ Merck, Darmstadt, Germany), with sample concentration of 1.0 mM and 0.1 M tetrabutylammonium perchlorate (*n*-Bu_4_NClO_4_) as the supporting electrolyte. A standard three-electrode cell was used, with a 3 mm diameter glassy carbon working electrode, a platinum wire counter electrode and an Ag/AgCl coupled with a Luggin capillary reference electrode. All potentials were referred to the redox potential of ferrocene (Fc)/ferrocenium ion (Fc^+^) as an internal reference (Figures S3 and S4).

### 3.2. Synthesis of [n-Bu_4_N]_2_[Mo_6_O_18_NC_6_H_4_N_3_C_2_H_2_] (***1***)

A mixture of [*n*-Bu_4_N]_2_[Mo_6_O_19_] (1.3636 g, 1.0 mmol), 4-(1*H*-1,2,4-Triazol-1-yl)aniline (0.1762 g, 1.1 mmol), and *N*,*N*′-dicyclohexyl carbodiimide (DCC) (0.2682 g, 1.3 mmol) was added to 20 mL anhydrous acetonitrile under a N_2_ gas flow and refluxed for 24 h. During the course of the reaction, the colour of the solution changed from yellow to red, and some white precipitate (*N*,*N*′-dicyclohexylurea obtained via the decomposition of the DCC) was formed. After being cooled down to room temperature, the red solution was filtered to remove the white precipitate. The filtrate was poured into 150:50 mL of diethyl ether:ethanol obtaining a red precipitate. The solid was washed with ethanol, ethyl acetate, and diethyl ether several times and then dissolved in acetone. The red solution was filtered and concentrated with a rotary evaporator. Single crystals were obtained within five days in a tube by slow diffusion of diethyl ether into an acetone solution of the crude product. (See Supporting Information, Figure S1) Yield: 0.1250 g, ≈8.3% based on Mo. Elemental Analysis: Calculated (experimental) for C_40_H_78_Mo_6_N_6_O_18_: C: 31.89% (32.03); N: 5.58% (5.39); H: 5.22% (6.91) FTIR-ATR (cm^−1^): 2959(w), 2933(w), 2871(w), 1507(m), 1474(m), 1462(w), 1379(w), 1331(w), 1277(w), 1213(w), 1140(w), 1104(vw), 1046(w), 1025(vw), 974 (m, shoulder: characteristic of mono-organoimido-substituted hexamolybdate), 948(s), 882(w), 845(w), 771(vs) (Figure S7). UV-vis (CH_3_CN, nm): λ_max_ = 356. ESI-MS (CH_3_CN, *m*/*z*): 510.6 [Mo_6_O_18_NC_6_H_4_-N_3_C_2_H_2_]^2−^ (Figure S8).

### 3.3. Synthesis of [n-Bu_4_N]_2_[Mo_6_O_18_NC_13_H_9_] (***2***)

A mixture of [*n*-Bu_4_N]_2_[Mo_6_O_19_] (1.3636 g, 1.0 mmol), 2-Aminofluorene (0.1994 g, 1.1 mmol), and *N*,*N*′-dicyclohexyl carbodiimide (DCC) (0.2682 g, 1.3 mmol) was added to 20 mL anhydrous acetonitrile under a N_2_ gas flow and refluxed for 24 hours. During the course of the reaction, the colour of the solution changed from yellow to red, and some white precipitate (*N*,*N*′-dicyclohexylurea was obtained via the decomposition of the DCC) was formed. After being cooled down to room temperature, the red solution was filtered to remove the white precipitate. The filtrate was poured into 150:50 mL of diethyl ether:ethanol obtaining a red precipitate. The solid was washed with ethanol, ethyl acetate, and diethyl ether for several times and then dissolved in acetone. The red solution was filtered and concentrated with a rotary evaporator. Single crystals were obtained within seven days in a tube by slow diffusion of diethyl ether into an acetonitrile solution of the crude product. (See Supporting Information, Figure S2) Yield: Yield: 0.1200 g, ≈7.8% based on Mo. Elemental Analysis: Calculated (experimental) for C_45_H_81_Mo_6_N_3_O_18_: C: 35.38% (35.50); N: 2.75% (2.63); H: 5.34% (5.93) FTIR-ATR (cm^−1^): 2959(m), 2931(m), 2871(m), 1712(w) 1631(w), 1599(w), 1479(m), 1448(m), 1378(w), 1322(w), 1295(w), 1223(w), 1154(w), 1107(w), 1065(vw), 1026(vw), 975 (w, shoulder: characteristic of mono-organoimido-substituted hexamolybdate), 945(s), 880(w), 765(vs) (Figure S7). UV-vis (CH_3_CN, nm): λ_max_ = 386. ESI-MS (CH_3_CN, *m*/*z*): 522.1 [Mo_6_O_18_NC_13_H_9_]^2−^ (Figure S8).

### 3.4. Crystal Structure Determination

Single-crystal X-ray diffraction analyses of **1** and **2** were done on a Bruker Smart APEX2 system at 297 K and SAINT [26] was used for data reduction. The crystal structures were solved by direct methods using XS in SHELXTL [27]. Hydrogen atom positions were calculated after each cycle of refinement with SHELXL [28] using a riding model for each structure, with a C–H distance of 0.93 or 0.97 Å. *U*_iso_(H) values were set equal to 1.2*U*_eq_ of the parent carbon atom. For **1**, during the last stages of refinement, it was clear that the position of the nitrogen atoms in the 1,2,4-triazole ring of **1** ([Mo_6_O_18_NC_6_H_4_N_3_C_2_H_2_]^2−^) may have two possible conformations, A and B. Occupations were then refined (with the constraint of adding 1) and finally held constant at 50%/50% for A/B, respectively (Scheme S1). Additional crystallographic and refinement details are given in Table S1. Structural drawings were carried out with DIAMOND-3.2k, supplied by Crystal Impact [29]. Crystallographic data for the structure reported in this paper have been deposited with the Cambridge Crystallographic Data Centre as supplementary publication number CCDC-1577980 (**1**) and CCDC-1881932 (**2**).

### 3.5. Computational Details

#### 3.5.1. Prediction of UV-Vis Spectra

Geometry optimization for the all the systems studied in this work were done using DFT calculations. The hybrid PBE0 functional [30,31,32] and a triple−ζ basis set for H, C, N, and O atoms including a polarization function for non-hydrogen atoms were employed [33]. Prediction of the electronic spectrum was developed using the time-dependent density functional theory included in the Gaussian09 Code [34]. The hybrid functional of Perdew Burk and Ernzerhof, PBE0, was used to represent the electron density. We have chosen a hybrid functional for the calculations instead of a GGA (Generalized Gradient Approximation) BP86 (Becke-Perdew 86) [35] because the HOMO–LUMO gap will be under-estimated due to the lack of a Hartree-Fock exchange. Therefore, the calculated electronic spectra will not be representative. For the Mo centres, the Stuttgart–Dresden core effective potential was employed to represent the orbital basis of these centres [36]. The continuum SMD (Solvation Model based on Density) model was employed to simulate the solvation effect including geometry relaxation [37]. All relaxed structures obtained were validated by vibrational analysis. In all calculations, 150 singlet excitations were considered. The more intense excitations were treated using the hole–electron framework [24] by means of the MULTIWFN3.6 package [38]. Orbital and electronic density surfaces were plotted using isovalues of 0.05 and 0.001 atomic units, respectively.

#### 3.5.2. Theoretical Redox Potentials

All calculations were carried out using the conceptual framework of density functional theory included in the Gaussian09 Code [34]. An open-shell spin-unrestricted approach was considered to describe the electron density, using the hybrid functional B3LYP (Becke, three-parameter, Lee-Yang-Parr) [39]. To represent H, C, N, and O atoms, a tripleε−ζ orbital basis set including a polarization function for non-hydrogen atoms was employed [33]. In the case of Mo centres, the relativistic pseudo-potential proposed by Hay and Wadt LANL2DZ (Los Alamos National Laboratory 2-double-z) was used [40]. The continuum universal solvation model developed by Thrular (SMD) was employed to represent the acetonitrile solvation effect, and was considered for all geometry optimization processes and single point calculations [37]. All structures were validated by means of their vibrational analyses. Adiabatic redox potentials were calculated from solvated geometries considering the direct step as recommended by Poblet et al. [14].

(1) Ox(solv)+e(solv)→Red(solv)

The Redox potentials were obtained from Equation (2) considering an only one–electron reaction:

(2) $${\Delta G}_{\mathrm{redox}}={\mathrm{nFE}}_{\mathrm{redox}}$$

where ΔG_redox_ is the Gibbs free energy change of the redox reaction, *n* is the number of electrons transferred in the reaction, F is Faraday´s constant, and E_redox_ is the redox potential. To refer the potential to the ferrocene/ferrocenium process, these redox potentials were calculated with the same method, i.e., as an adiabatic redox potential considering relaxed geometries in solution.

## 4. Conclusions

For aliphatic substituents a more *sp* nitrogen atom is observed, therefore it interacted as a triple bond to the molybdenum atom and single bond to the substituent. On the other hand, for the aromatic substitution, the interaction presented less of a triple-bond character to the molybdenum, and an increased double-bond interaction to the substituent.

For all the organo-imido studied systems, the aliphatic fragments compared to the aromatic ones had a bigger influence in the electrochemical properties as observed using the cathodic shift parameter.

The spectroscopic properties of all studied organo-imido compounds showed that the aromatic substitution with a higher conjugation could shift to higher energies through the O.F. to Mo CT compared to the aliphatic substituents.

The CT band was almost not affected by the coordination of a metal complex connected to organo-imido POM.

No relation between the wavelength of both CT transitions and the theoretical and experimental cathodic shift was observed, suggesting that the orbitals involved in each process were not equally affected upon functionalization (Figure S9).

## Figures and Tables

**Table 1:** Summary of the Mo–N–C angle that connects the organic fragment and the polyoxometalate unit.

Mo_6_O_18_N-R	Mo–N–C Angle (°)	Ref.
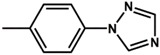 **1**	159.8(5)	This work
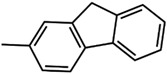 **2**	158.1(2)	This work
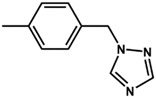 **3**	163.5(2)/164.1(1)	[12]
–CH_2_CH_3_	178.1(1)	[21]
–CH_2_CH_2_CH_3_	177.9(5)	[21]
–CH_2_CH_2_CH_2_CH_3_	178.3(8)	[21]
–CH_2_CH_2_CH_2_CH_2_CH_2_CH_3_	176.7(1)	[21]

**Table 2:** Summary of experimental (ΔE_1/2_) and theoretical (ΔE_C_) cathodic shifts for the first reduction potential of hexamolybdate functionalized with different organo-imido groups. Also, the experimental E_1/2_ of **1**, **2**, **3**, and **4** are presented.

Mo_6_O_18_N–R	ΔE_1/2_ (V)	ΔE_C_ (V)	E_1/2_ (V)	E_1/2-Teo_ (V)	Ref.
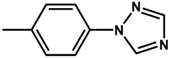 **1**	0.190	0.286	−1.026	-0.973	This work
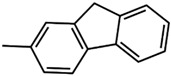 **2**	0.183	0.308	−1.019	−0.995	This work
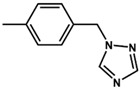 **3**	0.175	0.252	−1.011	−0.939	[12]
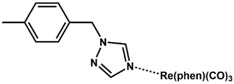 **4**	0.130	0.224	−0.966	−0.910	[12]
–CH_3_	0.270	0.341		−1.028	[21]
–CH_2_CH_3_	0.320	0.340		−1.020	[21]
–CH_2_CH_2_CH_3_	0.258	0.278		−0.964	[21]
–CH_2_CH_2_CH_2_CH_3_	0.288	0.324		−1.011	[21]
–CH_2_CH_2_CH_2_CH_2_CH_2_CH_3_	0.270	0.306		−0.993	[21]
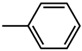	0.190	0.286		−0.973	[23]
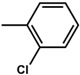	0.191	0.237		−0.924	[22]
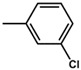	0.197	0.307		−0.994	[22]
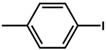	0.161	0.219		−0.906	[10]
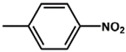	0.119	0.169		−0.856	[10]

**Table 3:** Hole-electron surfaces and summary of calculated wavelengths (O.F. → Mo) and oscillator factors (f) of organic fragment to metal charge transfers”.

Mo_6_O_18_N–R	λ O.F. → Mo (nm)	f	References
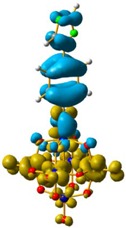 **1**	375	0.2951	This work
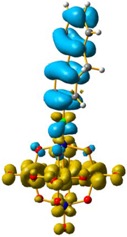 **2**	389	0.5770	This work
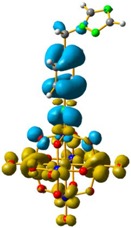 **3**	346	0.3772	[12]
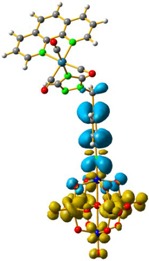 **4**	345	0.3976	[12]
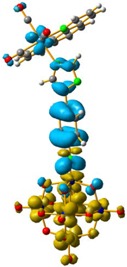 **5**	366	0.5993	This work
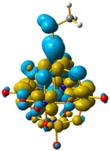 **6**	328	0.0100	[21]
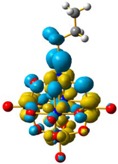 **7**	328	0.0119	[21]
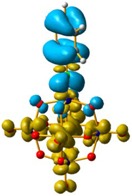 **8**	342	0.3901	[23]

## Supplementary Materials

The following are available online. X-Ray structures of 1 and 2 Figure S1. (a) Ball-and-stick representation of organic-inorganic hybrid ligand 1. (b) Hydrogen bond interactions, showing the connectivity between each organoimido-POM units. [*n*-Bu_4_N]^+^ ions are omitted for clarity. Scheme S1. Nitrogen location possibilities for the 1,2,4-triazole ring in 1. Figure S2. (a) Ball-and-stick representation of 2. (b) Crystalline lattice. [*n*-Bu_4_N]^+^ ions are omitted for clarity. Table S1. Crystal parameters and refinement details for 1 and 2. Figure S3. Cyclic voltamograms of 1 and 2, measured in CH_3_CN and *n*-NBu_4_ClO_4_ as the supporting electrolyte at a scan rate of 100 mV s^−1^. Figure S4. Comparison of the cyclic voltamograms of Mo_6_, [*n*-NBu_4_]_2_[Mo_6_O_19_] (black), 1 (blue), and 3 (red) measured in CH_3_CN and *n*-NBu_4_ClO_4_ as the supporting electrolyte at a scan rate of 100 mV s^−1^. All measurements are referred to the Fc/Fc^+^ potential. Figure S5. Overlap plot of the experimental spectra and oscillator strength calculated using TD-DFT methods, employing CH_3_CN as the continuum solvent for 2. Figure S6. Frontier orbitals surfaces (HOMO–LUMO) of systems 1, 2, 3, 4 and of the calculated structure of 5. Figure S7. FTIR-ATR spectrum in the 400 to 4000 cm^−1^ region of compound 1 (blue), 2 (red), and Mo_6_ (black). Figure S8. Experimental ESI-MS in CH_3_CN and theoretical isotopic patterns of 1 and 2. Figure S9. Relation between the charge transfer processes and the electrochemical processes. (a) Organic fragment to molybdenum charge transfer band vs. the experimental cathodic shift. (b) Oxygen to molybdenum charge transfer band vs. the experimental cathodic shift. (c) Organic fragment to molybdenum charge transfer band vs. the theoretical cathodic shift. (d) Oxygen to molybdenum charge transfer band vs. the theoretical cathodic shift.
