# Supplementary material for: Substitution Effect on the Charge Transfer Processes in Organo-Imido Lindqvist-Polyoxomolybdate
Source: Molecules. 2018 Dec 22;24(1):44. doi: 10.3390/molecules24010044 (PMC6337139; doi:10.3390/molecules24010044)
Supplement: Supplementary file 1 [file molecules-24-00044-s001.pdf]

Supporting Information

Substitution Effect on the Charge Transfer Processes in Organo-Imido Lindqvist-Polyoxomolybdate

Patricio Hermosilla-Ibáñez [1,2], Kerry Wrighton-Araneda [1,2], Walter Cañón-Mancisidor [1,2], Marlen Gutierrez-Cutiño [1,2], Verónica Paredes-García [2,3], and Diego Venegas-Yazigi [1,2,*]

[1] Facultad de Química y Biología, Universidad de Santiago de Chile, USACH; Av. Libertador Bernardo O'Higgins 3363, 9170022, Santiago, Chile; patricio.hermosilla@usach.cl (P.H.-I.); kerry.wrighton@usach.cl (K.W.-A.); walter.canon@usach.cl (W.C.-M.); marlen.gutierrez@usach.cl (M.G.-C.)
[2] Centro para el Desarrollo de la Nanociencia y Nanotecnología, CEDENNA, 9170022, Santiago, Chile
[3] Departamento de Ciencias Químicas, Universidad Andres Bello, Republica 275, 8370146, Santiago, Chile; vparedes@unab.cl (V.P.-G)
[*] Correspondence: diego.venegas@usach.cl (D.V.-Y.); Tel.: +56-22-718-1079

Figures

Crystal Structure [*n*-$Bu_4N]_2[Mo_6O_{18}NC_6H_4-N_3C_2H_2]$ (**1**)

Compound **1** crystallizes in a monoclinic system with the space group *P2₁/n*. The crystalline lattice is formed by the polyanion $[Mo_6O_{18}NC_6H_4-N_3C_2H_2]^{2-}$ and two [*n*-$Bu_4N]^+$ cations as charge compensating (Figure S1a). The bond distance Mo≡N is 1.735(4) Å, while Mo=O and Mo-O_{bridge} are in the range of 1.674(4) Å to 1.688(5) Å and 1.854(4) Å to 2.358(3) Å, respectively. The angle Mo1-N1-C1 is 159.8(5)° and the angles O-Mo=O and O-Mo-O are between 102.2(2)° to 179.6(2)° and 75.40(13)° to 153.58(16)°, respectively. The crystalline packing shows the spatial arrangement of the polyanionic units, these species are grouped dimers-type, these are stabilized mainly by hydrogen bond *C8A-H8A···O6^i^* ($d_{H\cdots O}$= 2.53 Å ; $d_{C\cdots O}$= 3.393(15) Å; *i*: 1-x,-y,1-z) (see Figure S1b).

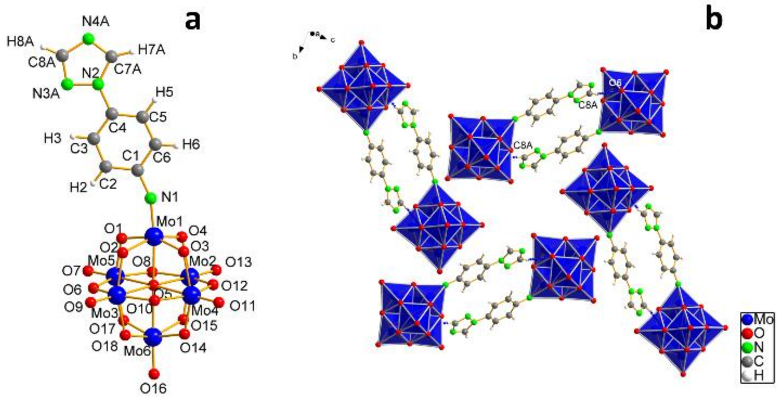

Figure S1. a) Ball-and-stick representation of organic-inorganic hybrid ligand **1**. b) Hydrogen bond interactions, showing the connectivity between each organoimido-POM units. [*n*-$Bu_4N]^+$ ions are omitted for clarity.

Crystal Structure [*n*-$Bu_4N]_2[Mo_6O_{18}NC_{13}H_9]$ (**2**)

Compound **2** crystallizes in an orthorhombic system with the space group *Pna2₁*. The crystalline lattice is formed by the polyanion $[Mo_6O_{18}NC_{13}H_9]^{2-}$ and two [*n*-$Bu_4N]^+$ cations as charge compensating. The bond distance Mo≡N is 1.710(2) Å, while Mo=O and Mo-O_{bridge} are in the range of 1.630(20) Å to 1.704(18) Å and 1.800(20) Å to 2.383(17) Å, respectively. The angle Mo1-N1-C1 is 158.1(18)° and the angles O-Mo=O and O-Mo-O are between 101.4(12)° to 179.1(10)° and 73.5(6)° to 154.8(7)°, respectively (Figure S2).

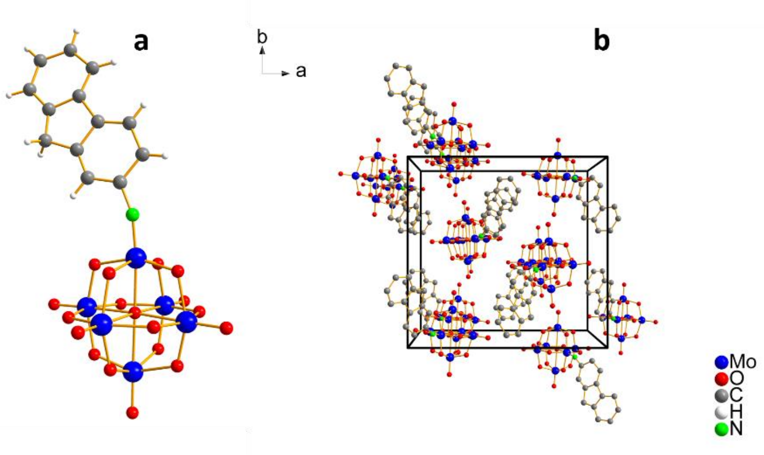

Figure S2. a) Ball-and-stick representation of **2**. b) Crystalline lattice. [n-$Bu_4N]^+$ ions are omitted for clarity.

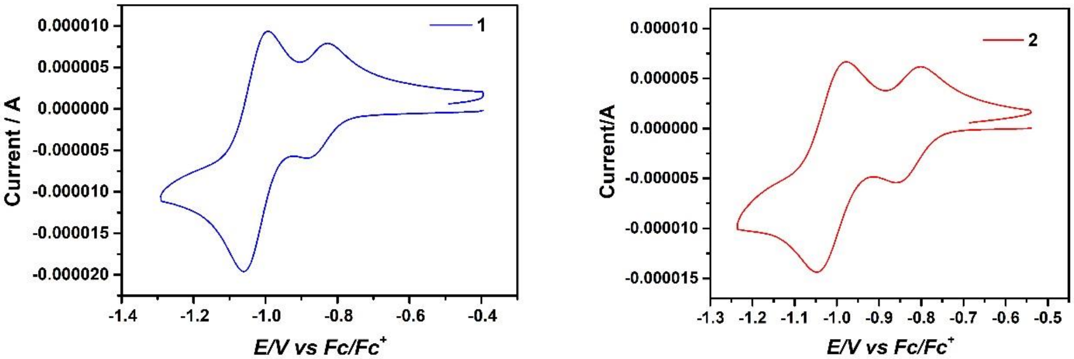

Figure S3. Cyclic voltammogram of compounds **1** and **2**, measured in CH_3CN and (n-Bu_4NClO_4) as the supporting electrolyte at scan rate of 100 mV/s. The potential was referred to Fc/Fc^+ couple. Compound **1** and **2** present oxidation potential at -0.991 V and -0.988 V, respectively. While, the reduction potential appears at -1,060 V and -1.050 V for **1** and **2**. The first wave that is observed correspond to oxidation a reduction of the hexamolybdate.

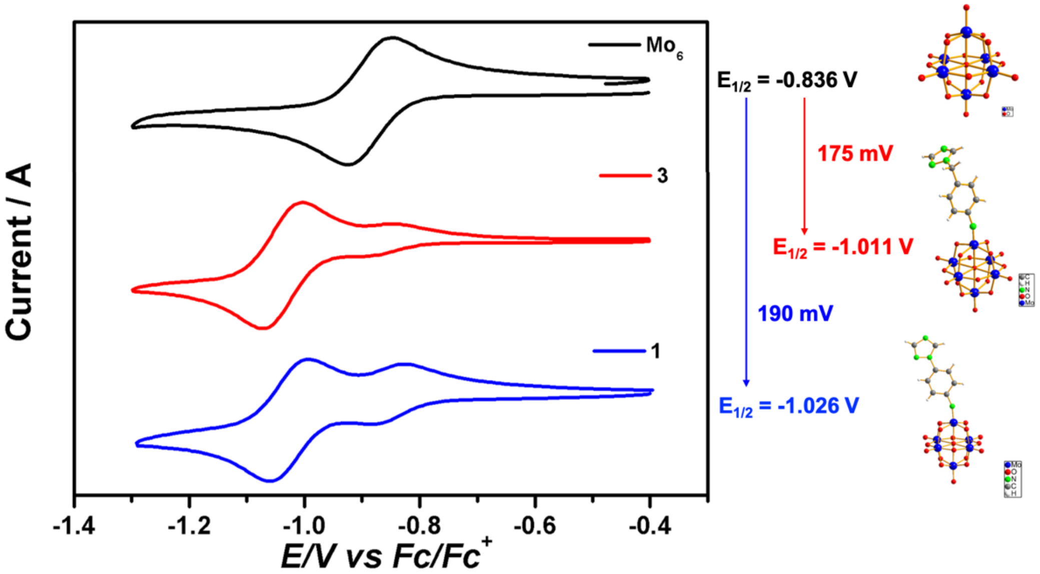

Figure S4. Comparison of the Cyclic Voltamograms of Mo_6, $[n\text{-}NBu_4]_2[Mo_6O_{19}]$ (black), **1** (blue) and **3** (red) measure in CH_3CN and n-NBu_4ClO_4 as the supporting electrolyte at a scan rate of 100 mV s^{-1}. All measurements are referred to the Fc/Fc^+ potential.

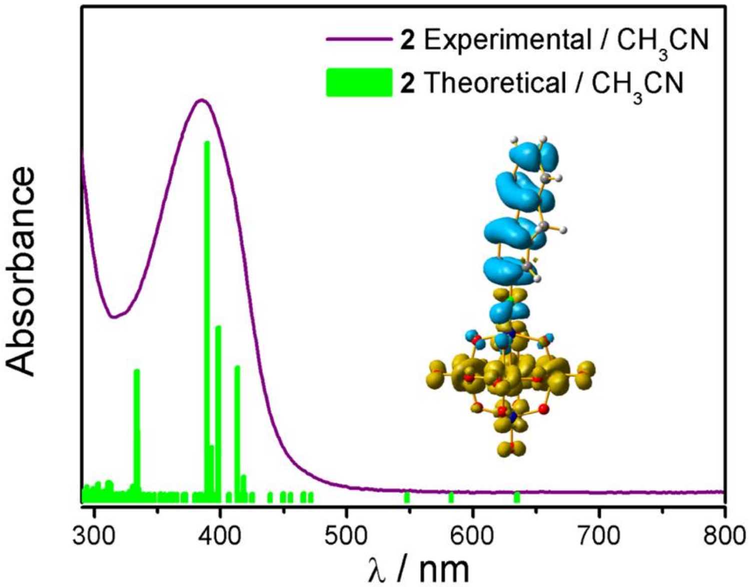

Figure S5. Overlap plot of the experimental spectra and oscillator strength calculated by TD-DFT methods, employing CH_3CN as the continuum solvent. Hole–electron density surface for the most intensive excitation of compound **2**. Light cyan and light brown represent the hole and electron surfaces, respectively.

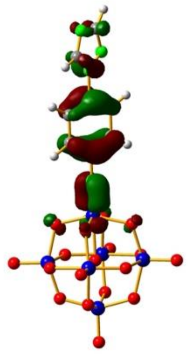

1-HOMO

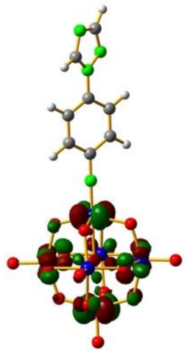

1-LUMO

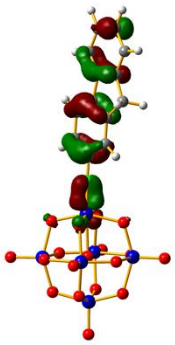

2-HOMO

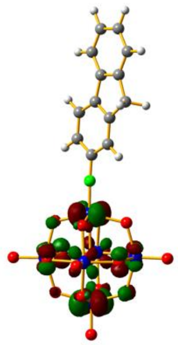

2-LUMO

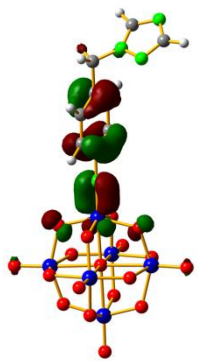

3-HOMO

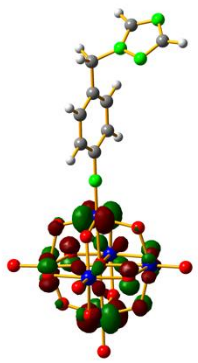

3-LUMO

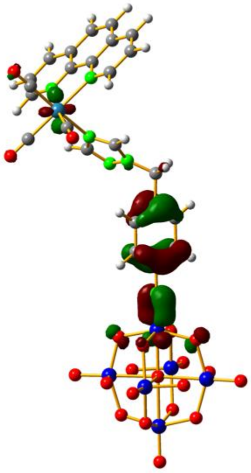

4-HOMO

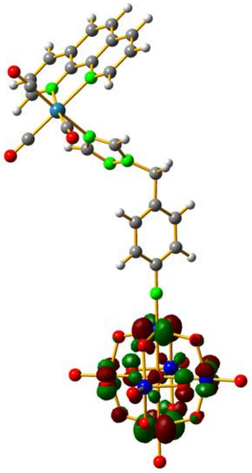

4-HOMO

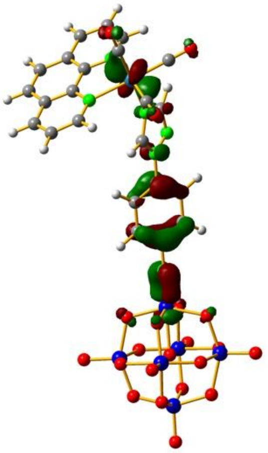

5-HOMO

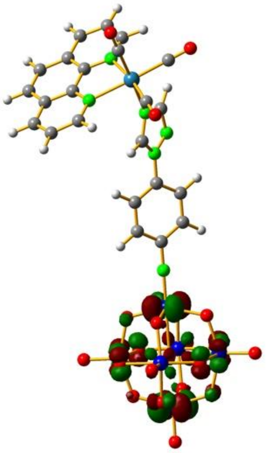

5-LUMO

Figure S6. Frontier orbitals surfaces (HOMO-LUMO) of systems **1**, **2**, **3**, **4** and of the calculated structure of **5**.

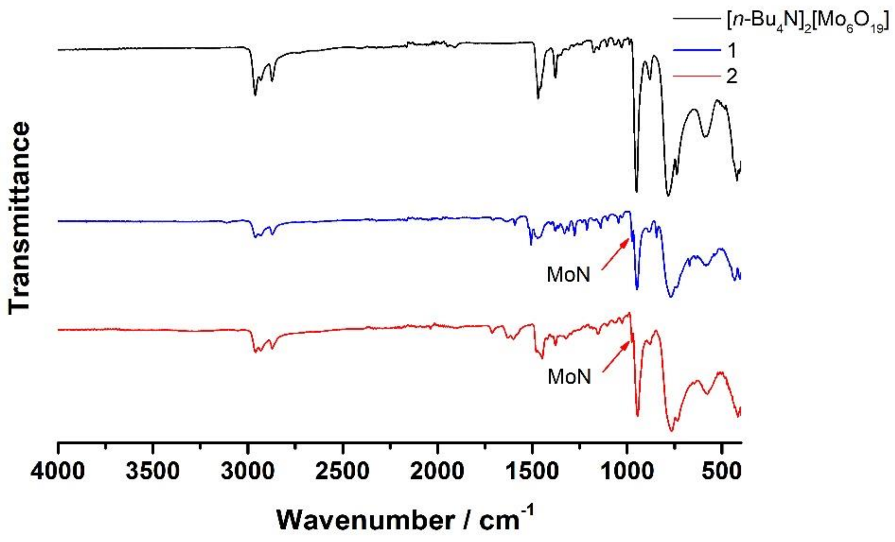

Figure S7. FTIR-ATR spectrum in the 400 to 4000 cm^{-1} region of compound **1** (blue) **2** (red) and Mo_6 (black).

The FTIR-ATR of **1** present the characteristic shoulder of mono-organoimido-substituted hexamolybdate at 974 cm^{-1} (νMo≡N), while the Mo=O stretching band appear at 948 cm^{-1} (Figure S3) and the asymmetric vibration mode Mo-O-Mo is observed at 770 cm^{-1}.

The FTIR-ATR of **2** present the characteristic shoulder of mono-organoimido-substituted hexamolybdate at 975 cm^{-1} (νMo≡N), while the Mo=O stretching band appear at 945 cm^{-1} (Figure S3) and the asymmetric vibration mode Mo-O-Mo is observed at 765 cm^{-1}.

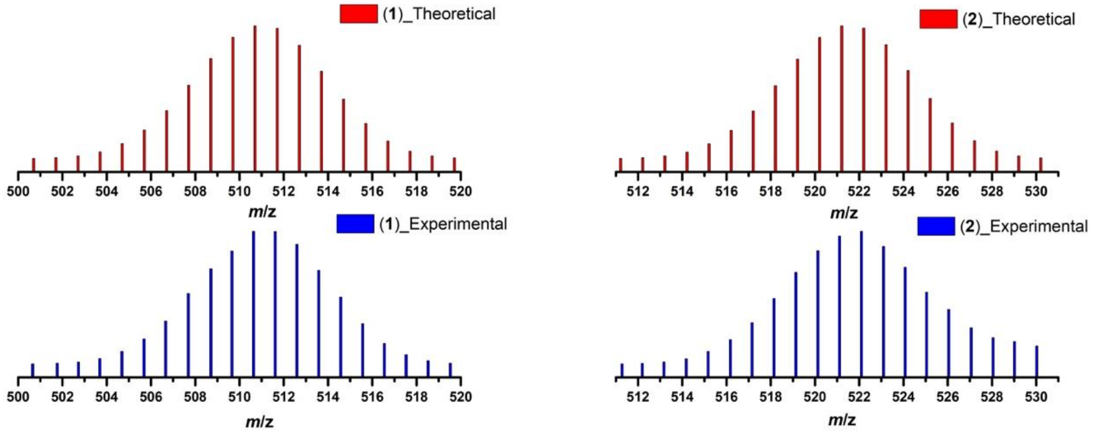

Figure S8. Experimental ESI-Mass spectrometry in CH_3CN and theoretical isotopic patterns of **1** and **2**.

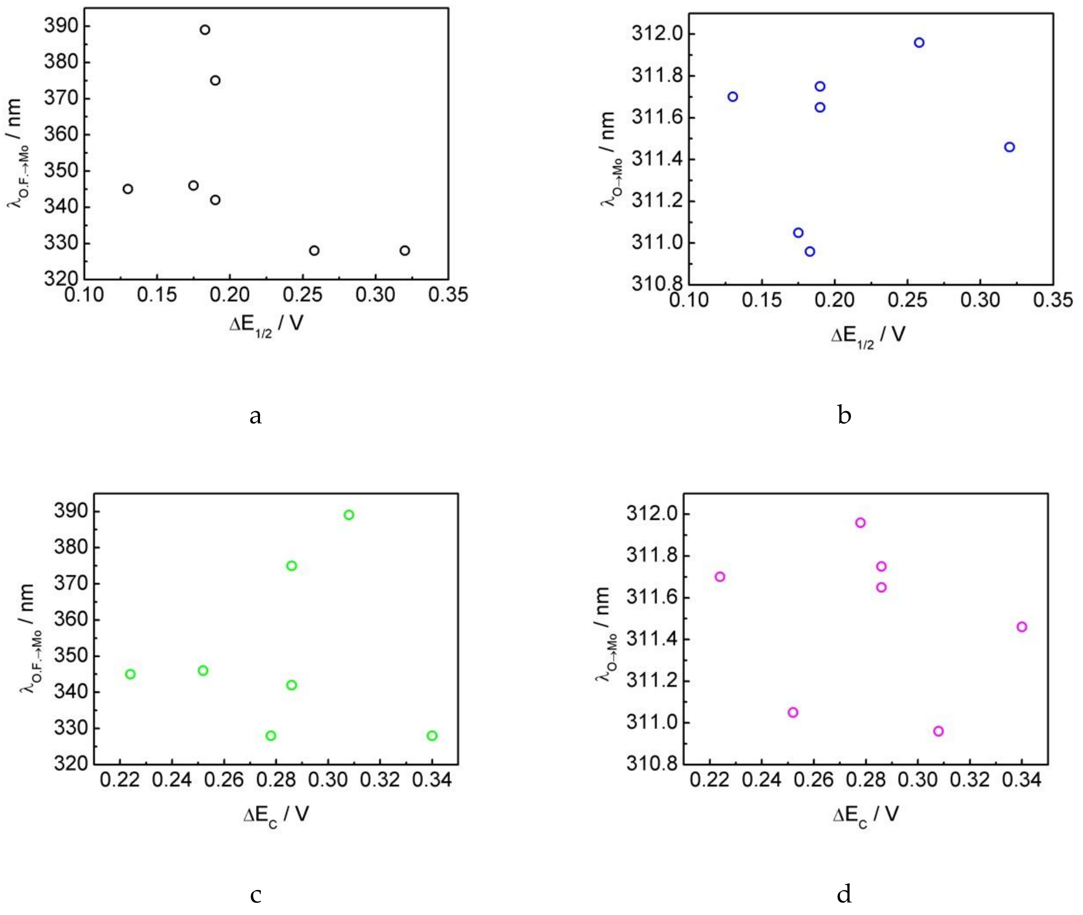

Figure S9. Relation between the charge transfer processes and the electrochemical processes. a. organic fragment to molybdenum charge transfer band vs the experimental cathodic shift. b. oxygen to molybdenum charge transfer band vs the experimental cathodic shift. c. organic fragment to molybdenum charge transfer band vs the theoretical cathodic shift. d. oxygen to molybdenum charge transfer band vs the theoretical cathodic shift.

.

Schemes

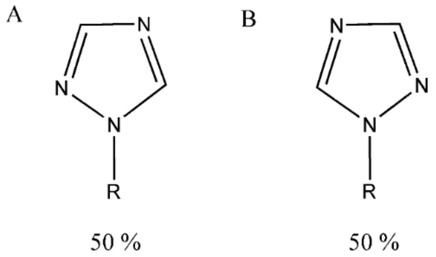

Scheme S1. Nitrogen location possibilities for the 1,2,4-triazole ring in **1**.

Tables

Table S1. Crystal parameters and refinement details for **1** and **2**

CCDC	1577980	1881932
Empirical Formula	$C_{40}H_{78}Mo_6N_6O_{18}$	$C_{45}H_{81}Mo_6N_3O_{18}$
Formula weight	1506.72	1527.77
Temperature/K	297(2)	297(2)
Crystal system	Monoclinic	Orthorhombic
Space group	$P2_1/n$	$Pna2_1$
a/Å	17.0724(12)	19.391(3)
b/Å	15.9575(12)	18.501(2)
c/Å	21.7898(16)	16.080(2)
β/°	104.5790(10)	-
Volume/Å^3	5745.1(7)	5768.5(13)
Z	4	4
ρ_{calc} g/cm^3	1.742	1.759
μ/mm^{-1}	1.338	1.332
F(000)	3024.0	3072.0
Crystal size/mm^3	0.23 × 0.21 × 0.17	0.30 × 0.30 × 0.24
Radiation	MoKα (λ = 0.71073)	MoKα (λ = 0.71073)
2Θ range for data collection/°	3.494 to 51.998	3.356 to 56.142
Index ranges	-20 ≤ h ≤ 21,<br>-19 ≤ k ≤ 19,<br>-26 ≤ l ≤ 26	-25 ≤ h ≤ 24,<br>-23 ≤ k ≤ 23,<br>-20≤ l ≤20
Reflections collected	44444	47390
Independent reflections	11272[R(int) = 0.0373]	12884[R(int) = 0.0667]
Data/restraints/parameters	11272/130/675	12884/255/657
Goodness-of-fit on F^2	1.037	0.958
Final R indexes [I>=2σ (I)]	R_1 = 0.0502, wR_2 = 0.1254	R_1 = 0.0831, wR_2 = 0.1990
Final R indexes [all data]	R_1 = 0.0781, wR_2 = 0.1458	R_1 = 0.1871, wR_2 = 0.2622
Largest diff. peak/hole / e Å^{-3}	1.06/-0.45	1.18/-0.47

Table S2. Summary of calculated wavelength ($\lambda_{O\rightarrow Mo}$) and oscillator strength (f) of oxygen to metal charge transfer

$Mo_6O_{18}N$-R	$\lambda_{O\rightarrow Mo}$ (nm)	f	References
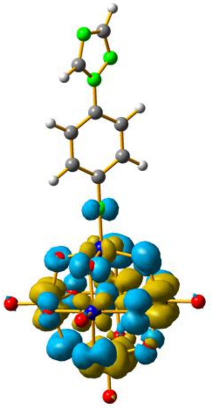 **1**	312	0.0236	This work
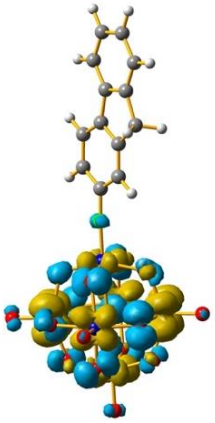 **2**	311	0.0200	This work
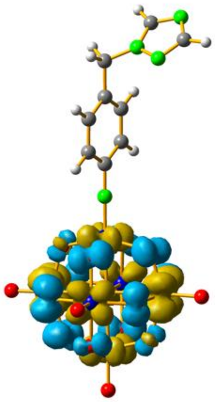 **3**	311	0.0397	[12]

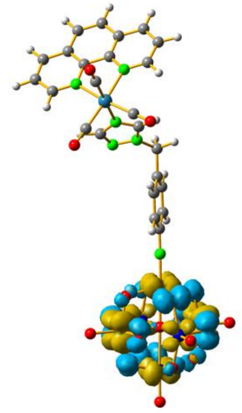 **4**	312	0.0336	[12]
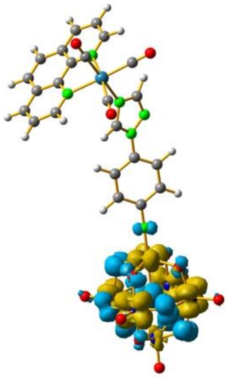 **5**	311	0.0352	This work
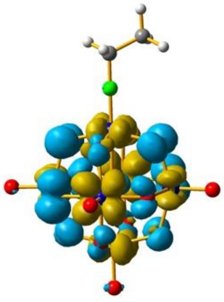 6	311	0.0330	[21]

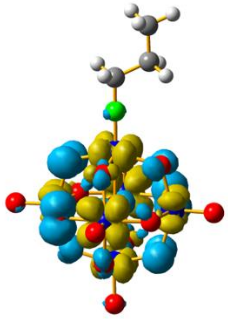<br>7	312	0.0334	[21]
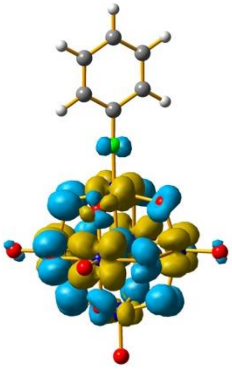<br>8	312	0.0267	[23]
